# Missing Globe: A Case of Severe Head Trauma, Eyelid Laceration, and Traumatic Enucleation

**DOI:** 10.7759/cureus.1988

**Published:** 2017-12-26

**Authors:** Joobin Khadamy, Pardis Khademi, Mohsen Kashkouli

**Affiliations:** 1 Eye Research Center, Rassoul Akram Hospital, Iran University of Medical Science (Iums); 2 St. Erik Eye Hospital, Stockholm, Sweden., St. Erik Eye Hospital; 3 Eye Research Center, Rassoul Akram Hospital, Iran University of Medical Science (iums), Tehran, Iran

**Keywords:** eyelid laceration, ocular injuries, traumatic enucleation, missing globe, optic nerve regeneration

## Abstract

We report an unusual periocular injury in a 19-year-old motorcycle rider during an accident. The patient had lacerations on the right upper and lower eyelids and the globe was enucleated en bloc. Despite disorganization of the eyelids and orbit, reconstruction of the eyelids and anophthalmic socket was successful. The primary reconstruction of the anophthalmic socket in the traumatic enucleation is a real challenge, especially when the conjunctival and the orbital tissues are missing or disorganized. It is proposed to utilize the optic nerve regeneration techniques in the cases of traumatic enucleation when the globe is intact. However, in the current case, the globe was unavailable.

## Introduction

Ocular injuries are common. Most of them are minor with no serious impact on the patients’ vision [1]. Globe luxation and the traumatic enucleation caused by high energy trauma are very uncommon [2-3]. Additionally, missing the globe at the accident site has not yet been reported.

We present a case of traumatic enucleation and eyelid laceration whose globe was missed on the site of the accident. The challenges that the surgeons may encounter in such a case are discussed. The informed consent was obtained from the patient. The report was approved by the ethics committee of the ophthalmology department.

## Case presentation

**Figure 1:**
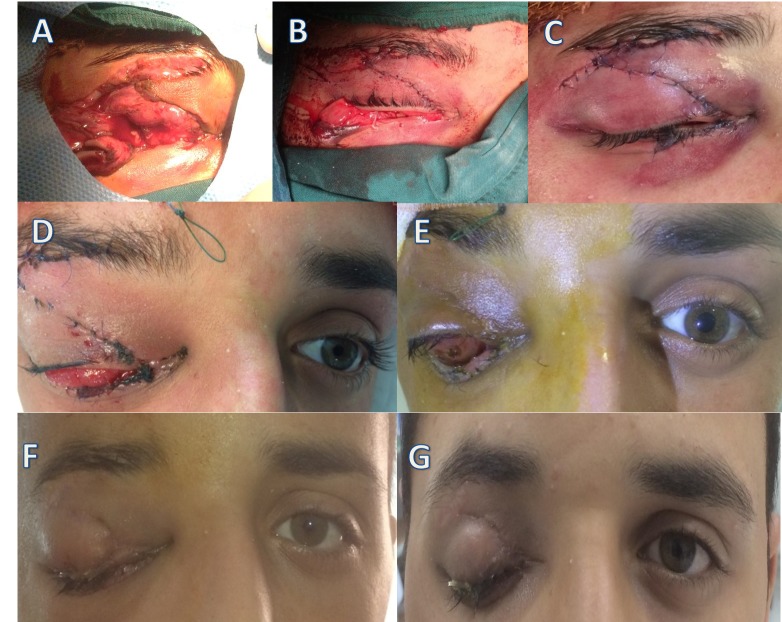
The pre-operative, intra-operative and post-operative images. A: The pre-operative view showing the extensive upper and lower lid lacerations. The orbital tissues are unremarkable. No sign of the globe was evident, B and C: After the cautious exploration of the orbit of any remnants of the globe, the conjunctiva, upper, and lower eyelids were repaired, D and E: The images were taken on the third and seventeenth post-operative days, respectively. Since the chemotic and short conjunctiva led to frequent extrusion of the conformer, a median tarsorrhaphy was performed, F and G: The images were taken in the third and seventh postoperative weeks. A median tarsorrhaphy and larger conformer are in place.

The emergency room informed the night-shift ophthalmologist about a case of head trauma, eyelid laceration and possible globe laceration in a patient under general anesthesia for the urgent neurosurgical intervention. The anesthesiologist suggested that the ophthalmic surgery could be performed during the same surgical session. The patient was a motorcycle rider who hit the back of a pickup truck. The patient had extensive upper and lower eyelid lacerations. The trauma created skin flaps on the right upper and lower eyelids. Upon examination, the right globe was not evident (Figure 1A).

**Figure 2:**
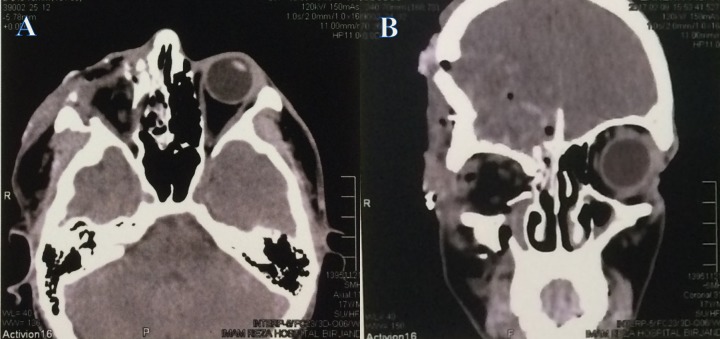
The computed tomography scans. A: An axial view of the orbital CT scans demonstrating the medial and lateral wall of the orbit was fractured. There were no clues that indicate the presence of a globe, B: The coronal view of the orbital CT scans revealed the four walls fracture of the right orbit. The frontal bone is extensively inferiorized. Pneumocephalus and severe adjacent brain injuries were evident. No sign of the right globe was visualized.

The computed tomography (CT) scan was reviewed. The four walls of the right orbit had fractures and were displaced. The frontal bone was extensively displaced downward. The orbital soft tissues were disorganized. No globe remnant was evident in the scan (Figure 2).

A cautious exploration of the orbit with fine forceps for any remnant of the globe was unsuccessful, blood covered the field, but cauterization was considered unsafe. Further gentle exploration of the orbit was performed. A cord-like tissue located was assumed to be the transected optic nerve, it was finally isolated. The visualization of a transected optic nerve confirmed that the globe was enucleated and missed at the accident scene.

The patient was admitted to the intensive care unit (ICU) afterward. The possibility of a future operation was not predictable. The reconstruction was a real challenge. Neither donor sclera nor amniotic membrane was available. The bulbar conjunctiva and globe were lost. The palpebral conjunctiva was shredded. The conjunctiva and other layers were blended. Landmarks could not be easily isolated. The conjunctival tissue was too short to reconstruct the anophthalmic socket properly. A conjunctiva autograft from the contralateral healthy eye was deemed unwise. A dermis fat or mucosal graft could have been possible, but was not tried, as these might prolong the operating time, and this was an emergency case.

Unlike a usual reconstruction, the palpebral conjunctiva was first sutured with absorbable sutures to help reveal the palpebral conjunctiva and its continuity on the fornices. Next, the eyelid margins, tarsal plate, and orbicularis layers were reconstructed in the standard manner. Finally, the upper and lower forniceal conjunctivas were sutured together (shown in Figure 1B-1C). A conformer was placed to expand the fornices. This was done to save the maximum possible forniceal conjunctiva for the future implant fitting.

During follow up, the patient complained of frequent extrusion of the conformer which was believed to be due to inadequate forniceal space as well as chemosis (shown in Figure 1D-1E). To expand the conjunctival surface, a larger conformer was inserted, followed by a temporary median tarsorrhaphy to prevent further extrusions (shown in Figure 1F-1G).

## Discussion

This is the first reported case of a traumatic enucleation with the missing globe. The traumatic enucleation may be caused by a retrobulbar posterior-to-anterior force. This leads to globe luxation. A sudden synchronized cutting force is needed to transect the optic nerve. It could be due to a retrobulbar foreign body or inward displacement of the orbital walls by high-energy trauma [2-3]. Paul, et al. reported a patient falling on a door stop that caused optic nerve transection and extrusion of the globe [3]. The eyeball was still in the orbit, opening in that case.

In the current case, the globe was pushed forward by decreased orbital volume resulting from inferiorized frontal bone and lateralized medial orbital bones (shown in Figure 2). However, the globe luxation alone is not enough, a sharp or sudden high-speed blunt trauma was needed to cut away both the eyelid and the optic nerve.

The most important consequence of such a trauma is the visual loss. However, the optimal primary repair is to restore the eyelid contours and also to provide an ideal anophthalmic socket is necessary for the future ocular prosthesis implant. This has considerable effects on the cosmesis and the psychological status of the patients. One may encounter the following challenges in the management of such cases. Further damage to a disorganized globe during the exploration should be avoided. If it is found that the globe is partly available, then ensuring that all remnants of the globe are excised is important as this may reduce the risk of sympathetic ophthalmia [4]. If the globe is intact, then the scleral tissue could be used to implant hydroxyapatite or sphere balls. The clinical applicability of this should be assessed. The reconstruction of the ocular surface and the alignment of the disorganized layers is a matter of patience, anatomical expertise, and surgical experience. Whether the optic nerve can be sutured or repaired to restore the vision in such eyes are unknown. There is currently no report of successful repair of the traumatic transected optic nerve in the human subjects; the data are limited to an animal subject report [5]. It has been proposed to attempt to utilize the optic nerve regeneration techniques in cases of the traumatic enucleation when the globe is intact. In traumatic enucleation cases, the utilization of the healthy fellow eye conjunctival autograft, amniotic membranes, and the mucosal tissue or dermis-fat grafts to reconstruct the anophthalmic socket merits requires further investigation.

## Conclusions

A case involving traumatic enucleation with the missing globe is presented. The primary reconstruction of an anophthalmic socket with the traumatic enucleation is a real challenge, especially when the conjunctival and the orbital tissues are missed and muddled. The challenges regarding the management of such cases should be noted. In traumatic enucleation cases that have intact globes, we suggest that the optic nerve regeneration techniques should be employed and that the visual and anatomical outcomes should be investigated. This could be a unique in vivo opportunity to investigate the possibility of the optic nerve regeneration and the autograft whole globe transplantation.
